# Supplementary material for: Climatic and soil characteristics account for the genetic structure of the invasive cactus moth Cactoblastis cactorum, in its native range in Argentina
Source: PeerJ. 2024 Feb 12;12:e16861. doi: 10.7717/peerj.16861 (PMC10868523; doi:10.7717/peerj.16861)
Supplement: Supplementary file 1 [file peerj-12-16861-s001.doc]

	1	2	3	4	5	6	7	8	9	10	11	12	13	14	15	16	17	18	19	20	21	22	23	24
**1**	0	0.2014	0.2154	0.2715	0.1996	0.1766	0.2482	0.1786	0.2195	0.1654	0.2111	0.2743	0.2178	0.2532	0.2435	0.2227	0.0733	0.1015	0.1559	0.0697	0.0753	0.1358	0.0705	0.1234
**2**	0.2014	0	0.1057	0.0699	0.1362	0.0906	0.1836	0.0875	0.1366	0.125	0.1561	0.2588	0.1711	0.2047	0.2233	0.1563	0.2013	0.1298	0.1754	0.1995	0.1912	0.096	0.1565	0.1069
**3**	0.2154	0.1057	0	0.2252	0.1206	0.0567	0.1958	0.1018	0.2047	0.1417	0.2112	0.294	0.2284	0.254	0.2044	0.2248	0.2335	0.1176	0.1408	0.2186	0.2225	0.097	0.1608	0.0881
**4**	0.2715	0.0699	0.2252	0	0.1498	0.1817	0.2368	0.1548	0.1587	0.1823	0.2304	0.3011	0.1915	0.265	0.2975	0.2011	0.2559	0.192	0.2598	0.2535	0.2318	0.1757	0.2439	0.1951
**5**	0.1996	0.1362	0.1206	0.1498	0	0.1454	0.2111	0.1054	0.144	0.111	0.1665	0.2235	0.1452	0.2211	0.2122	0.1848	0.2226	0.0879	0.1151	0.1857	0.2016	0.1128	0.1714	0.096
**6**	0.1766	0.0906	0.0567	0.1817	0.1454	0	0.1721	0.0776	0.1599	0.0907	0.1927	0.2659	0.1852	0.2178	0.1725	0.1929	0.198	0.0851	0.1269	0.168	0.1899	0.0593	0.1226	0.0687
**7**	0.2482	0.1836	0.1958	0.2368	0.2111	0.1721	0	0.1297	0.1314	0.1204	0.1593	0.2669	0.1728	0.236	0.1979	0.1966	0.2153	0.1351	0.2262	0.2148	0.2113	0.1269	0.1834	0.1623
**8**	0.1786	0.0875	0.1018	0.1548	0.1054	0.0776	0.1297	0	0.0909	0.074	0.1154	0.1874	0.124	0.1673	0.153	0.0881	0.1641	0.0714	0.1307	0.175	0.1612	0.0456	0.1449	0.0789
**9**	0.2195	0.1366	0.2047	0.1587	0.144	0.1599	0.1314	0.0909	0	0.102	0.0932	0.1277	0.054	0.1362	0.1863	0.0672	0.1884	0.0954	0.2006	0.2043	0.1818	0.0999	0.1989	0.1414
**10**	0.1654	0.125	0.1417	0.1823	0.111	0.0907	0.1204	0.074	0.102	0	0.1289	0.1928	0.1119	0.1605	0.1726	0.1299	0.1752	0.0695	0.1267	0.1393	0.1735	0.0571	0.131	0.0669
**11**	0.2111	0.1561	0.2112	0.2304	0.1665	0.1927	0.1593	0.1154	0.0932	0.1289	0	0.1401	0.0818	0.1315	0.167	0.1272	0.1974	0.1028	0.1979	0.223	0.1968	0.1166	0.2162	0.1201
**12**	0.2743	0.2588	0.294	0.3011	0.2235	0.2659	0.2669	0.1874	0.1277	0.1928	0.1401	0	0.0715	0.0961	0.2587	0.1415	0.2719	0.1501	0.2581	0.2663	0.2558	0.1855	0.2881	0.1943
**13**	0.2178	0.1711	0.2284	0.1915	0.1452	0.1852	0.1728	0.124	0.054	0.1119	0.0818	0.0715	0	0.0699	0.1884	0.1027	0.2158	0.0957	0.188	0.1985	0.1924	0.1131	0.1936	0.1396
**14**	0.2532	0.2047	0.254	0.265	0.2211	0.2178	0.236	0.1673	0.1362	0.1605	0.1315	0.0961	0.0699	0	0.2375	0.155	0.255	0.14	0.246	0.2417	0.2412	0.1449	0.2348	0.168
**15**	0.2435	0.2233	0.2044	0.2975	0.2122	0.1725	0.1979	0.153	0.1863	0.1726	0.167	0.2587	0.1884	0.2375	0	0.2196	0.2437	0.146	0.2132	0.2248	0.2152	0.1531	0.2162	0.1424
**16**	0.2227	0.1563	0.2248	0.2011	0.1848	0.1929	0.1966	0.0881	0.0672	0.1299	0.1272	0.1415	0.1027	0.155	0.2196	0	0.2002	0.1229	0.1772	0.215	0.1721	0.0919	0.2104	0.1405
**17**	0.0733	0.2013	0.2335	0.2559	0.2226	0.198	0.2153	0.1641	0.1884	0.1752	0.1974	0.2719	0.2158	0.255	0.2437	0.2002	0	0.1357	0.2332	0.1202	0.0339	0.159	0.1316	0.1734
**18**	0.1015	0.1298	0.1176	0.192	0.0879	0.0851	0.1351	0.0714	0.0954	0.0695	0.1028	0.1501	0.0957	0.14	0.146	0.1229	0.1357	0	0.0922	0.1112	0.1222	0.0328	0.0891	0.0507
**19**	0.1559	0.1754	0.1408	0.2598	0.1151	0.1269	0.2262	0.1307	0.2006	0.1267	0.1979	0.2581	0.188	0.246	0.2132	0.1772	0.2332	0.0922	0	0.1501	0.2094	0.0664	0.1096	0.0537
**20**	0.0697	0.1995	0.2186	0.2535	0.1857	0.168	0.2148	0.175	0.2043	0.1393	0.223	0.2663	0.1985	0.2417	0.2248	0.215	0.1202	0.1112	0.1501	0	0.1026	0.1412	0.0289	0.1299
**21**	0.0753	0.1912	0.2225	0.2318	0.2016	0.1899	0.2113	0.1612	0.1818	0.1735	0.1968	0.2558	0.1924	0.2412	0.2152	0.1721	0.0339	0.1222	0.2094	0.1026	0	0.1531	0.1231	0.1695
**22**	0.1358	0.096	0.097	0.1757	0.1128	0.0593	0.1269	0.0456	0.0999	0.0571	0.1166	0.1855	0.1131	0.1449	0.1531	0.0919	0.159	0.0328	0.0664	0.1412	0.1531	0	0.0858	0.0228
**23**	0.0705	0.1565	0.1608	0.2439	0.1714	0.1226	0.1834	0.1449	0.1989	0.131	0.2162	0.2881	0.1936	0.2348	0.2162	0.2104	0.1316	0.0891	0.1096	0.0289	0.1231	0.0858	0	0.0927
**24**	0.1234	0.1069	0.0881	0.1951	0.096	0.0687	0.1623	0.0789	0.1414	0.0669	0.1201	0.1943	0.1396	0.168	0.1424	0.1405	0.1734	0.0507	0.0537	0.1299	0.1695	0.0228	0.0927	0
